# The vitamin D receptor is involved in the regulation of human breast cancer cell growth via a ligand-independent function in cytoplasm

**DOI:** 10.18632/oncotarget.15803

**Published:** 2017-03-01

**Authors:** Trupti Trivedi, Yu Zheng, Pierrick G.J. Fournier, Sreemala Murthy, Sutha John, Suzanne Schillo, Colin R. Dunstan, Khalid S. Mohammad, Hong Zhou, Markus J. Seibel, Theresa A. Guise

**Affiliations:** ^1^ Bone Research Program, ANZAC Research Institute, University of Sydney, Sydney, Australia; ^2^ Division of Endocrinology, Department of Medicine, Indiana University-Purdue University at Indianapolis, Indianapolis, Indiana, USA; ^3^ Department of Biomedical Engineering, University of Sydney, Sydney, Australia; ^4^ Department of Endocrinology and Metabolism, Concord Hospital, Concord, Sydney, Australia; ^5^ Biomedical Innovation Department, Scientific Research and High Education Center from Ensenada (CICESE), Ensenada, Baja California, Mexico

**Keywords:** breast cancer, vitamin D, vitamin D receptor, ligand independent, bone metastasis

## Abstract

Vitamin D has pleiotropic effects on multiple tissues, including malignant tumors. Vitamin D inhibits breast cancer growth through activation of the vitamin D receptor (VDR) and via classical nuclear signaling pathways. Here, we demonstrate that the VDR can also function in the absence of its ligand to control behaviour of human breast cancer cells both outside and within the bone microenvironment. Stable shRNA expression was used to knock down VDR expression in MCF-7 cells, generating two VDR knockdown clonal lines. In ligand-free culture, knockdown of VDR in MCF-7 cells significantly reduced proliferation and increased apoptosis, suggesting that the VDR plays a ligand-independent role in cancer cell growth. Implantation of these VDR knockdown cells into the mammary fat pad of nude mice resulted in reduced tumor growth *in vivo* compared with controls. In the intra-tibial xenograft model, VDR knockdown greatly reduced the ability of the cells to form tumors in the bone microenvironment. The *in vitro* growth of VDR knockdown cells was rescued by the expression of a mutant form of VDR which is unable to translocate to the nucleus and hence accumulates in the cytoplasm. Thus, our data indicate that in the absence of ligand, the VDR promotes breast cancer growth both *in vitro* and *in vivo* and that cytoplasmic accumulation of VDR is sufficient to produce this effect *in vitro*. This new mechanism of VDR action in breast cancer cells contrasts the known anti-proliferative nuclear actions of the VDR-vitamin D ligand complex.

## INTRODUCTION

Breast cancer continues to be the most prevalent and frequently diagnosed malignancy in women worldwide [1]. In the past two decades, advances in early diagnostic and treatment strategies have decreased overall breast cancer mortality by more than 30% [2, 3]. However, once metastases have developed, the prognosis is poor [4–7].

Epidemiological and pre-clinical studies indicate an inverse correlation between serum concentrations of vitamin D and the development and progression of breast cancer [8–10]. Vitamin D deficiency in patients with early stage breast cancer is also associated with a poor prognosis [11].

The active metabolite of vitamin D, 1,25-dihydroxy-vitamin D_3_ (1,25D_3_) is a steroid hormone with pleiotropic effects on multiple tissues including bone, the immune system and cancer cells [12, 13]. The effects of vitamin D are mediated through binding of 1,25D_3_ to the vitamin D receptor (VDR). Upon ligand binding, the VDR forms a heterodimer complex with the retinoic acid receptor (RXR), which facilitates nuclear translocation and subsequent binding of the complex to specific vitamin D response elements (VDRE) within the promoters of target genes [14–17]. Well established ligand-mediated nuclear actions of the VDR include inhibition of cancer cell proliferation, induction of apoptosis, and inhibition of metastasis by regulating cell migration and invasion [15, 18–21].

Of note, VDR expression also negatively correlate with tumor progression: relatively high VDR concentrations are found in non-malignant hyperplastic tissue while progression to malignant growth is often associated with a reduction or complete loss of VDR expression [18, 22, 23]. Consistent with these reports, invasive breast cancers have been found to have relatively low VDR expression compared to normal mammary epithelia [22, 24]. Finally, VDR expression in breast cancer tissues may be inversely associated with the prognosis and survival of patients with breast cancer [25]. Collectively, these data suggest that vitamin D action through VDR in breast epithelial cells, which may act to prevent a switch toward tumorigenicity.

Previous studies in rodents have demonstrated that vitamin D deficiency promotes the growth of human breast cancer cells in bone [26, 27]. Using a vitamin D deficiency mouse model, these studies have shown that the acceleration in cancer cell growth was in part due to the effects of vitamin D deficiency on the bone microenvironment, as hypovitaminosis D induced a significant increase in osteoclast-mediated bone resorption due to secondary hyperparathyroidism [26, 28]. To further determine the interaction between tumor cells and vitamin D, bone resorption was inhibited through treatment with osteoprotegrin (OPG) in both vitamin D deficient and control mice. Interestingly, tumor burden in OPG-treated mice remained increased in vitamin D deficient mice relative to controls. It is thus likely that under these circumstances the absence of vitamin D had a direct effect on breast cancer growth [26, 27], which in turn poses the question as to whether vitamin D exerts direct inhibitory effects on cancer cell growth.

To determine the molecular mechanism of these direct effects, we disrupted vitamin D signaling in cancer cells and studied tumor growth in bone. Based on the known anti-proliferative function of vitamin D signaling, we hypothesized that silencing VDR expression in breast cancer cells would *promote* breast cancer growth. We therefore knocked down VDR expression in the human breast cancer cell line MCF-7 and followed up with clonal selection to generate highly efficient knockdown clones. In contrast to our initial hypothesis, we discovered that VDR knockdown inhibited cancer cell proliferation in the absence of vitamin D, suggesting a novel function of the VDR in promoting breast cancer cell growth.

## RESULTS

### Generation of stable VDR knockdown clones

**Figure 1:**
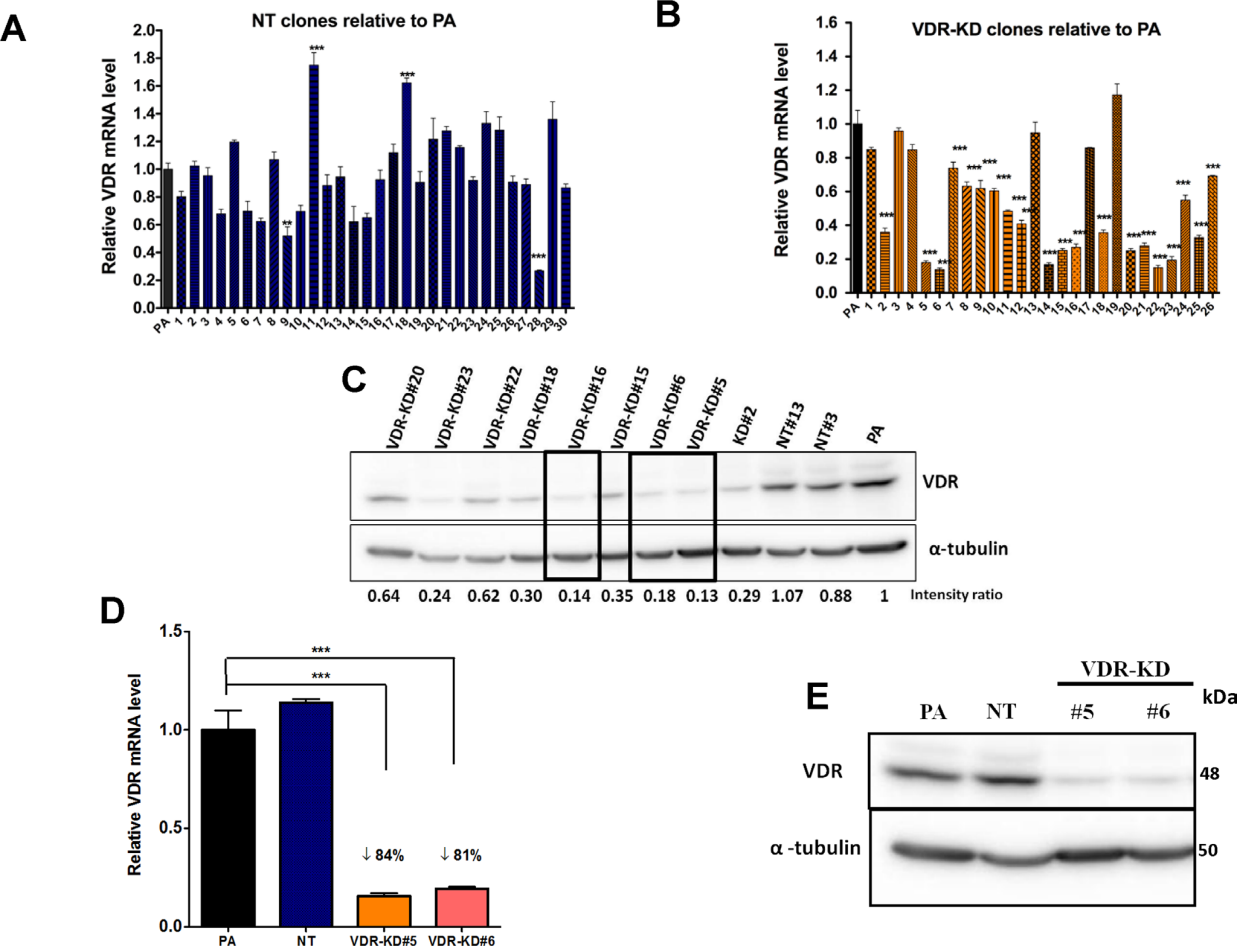
Stable knockdown of VDR in MCF-7 cells MCF-7 cells were transduced with a lentivirus expressing either a non-target shRNA (NT) or shRNA against VDR (VDR-KD) and single cell clones were selected. (**A**–**B**) Level of expression of VDR mRNA was measured using quantitative RT-PCR in (A) MCF-7 NT clones and (B) MCF-7 VDR-KD clones and compared to parental MCF-7 cells (PA). (**C**) Level of VDR protein in cell lysates of PA, NT and VDR-KD clones was assessed using Western blotting. (**D**–**E**) MCF-7 PA cells and NT and VDR-KD clones were grown for 8 weeks in absence of antibiotic and VDR mRNA and protein levels were reassessed to ensure stable knockdown. Results are expressed as the mean ± SEM (*n* = 3). ***p < 0.001 using one-way ANOVA with Tukey's post-test.

Parental MCF-7 cells were transduced with either the shVDR or shNT construct, then continuously maintained with complete media containing puromycin and allowed to grow exponentially before being used for single cell clonal selection. Out of 30 NT clones, NT#13 expressed VDR mRNA and protein levels similar to PA (Parental MCF-7) cells (Figure 1A, 1C) and was therefore selected for all subsequent experiments.

Out of 27 VDR-KD clones screened, clones #5, 6 and 16 exhibited knockdown of both VDR mRNA and protein expression between 80–85% compared to PA cells and NT clones (Figure 1B, 1C). Clones were retested for stability of VDR knockdown after culture in the absence of puromycin for 8 weeks. After 8 weeks, out of 3 clones, VDR knockdown in clones #5 and #6 remained stable both at mRNA and protein levels and were used for further experiments (Figure 1D, 1E). The overall level of VDR gene knockdown among the different VDR-KD clones is ∼50%, which may be due to variability within puromycin-resistant populations. The average of VDR mRNA levels of all VDR-KD clones was significantly reduced as compared to the average of VDR mRNA levels of all VDR NT clones (Mean ± SEM: 0.961 ± 0.0575 relative VDR mRNA in NT clones versus 0.515 ± 0.0553 relative VDR mRNA in VDR-KD clones, *p* < 0.001).

### VDR knockdown abrogates vitamin D signaling in MCF-7 cells

**Figure 2:**
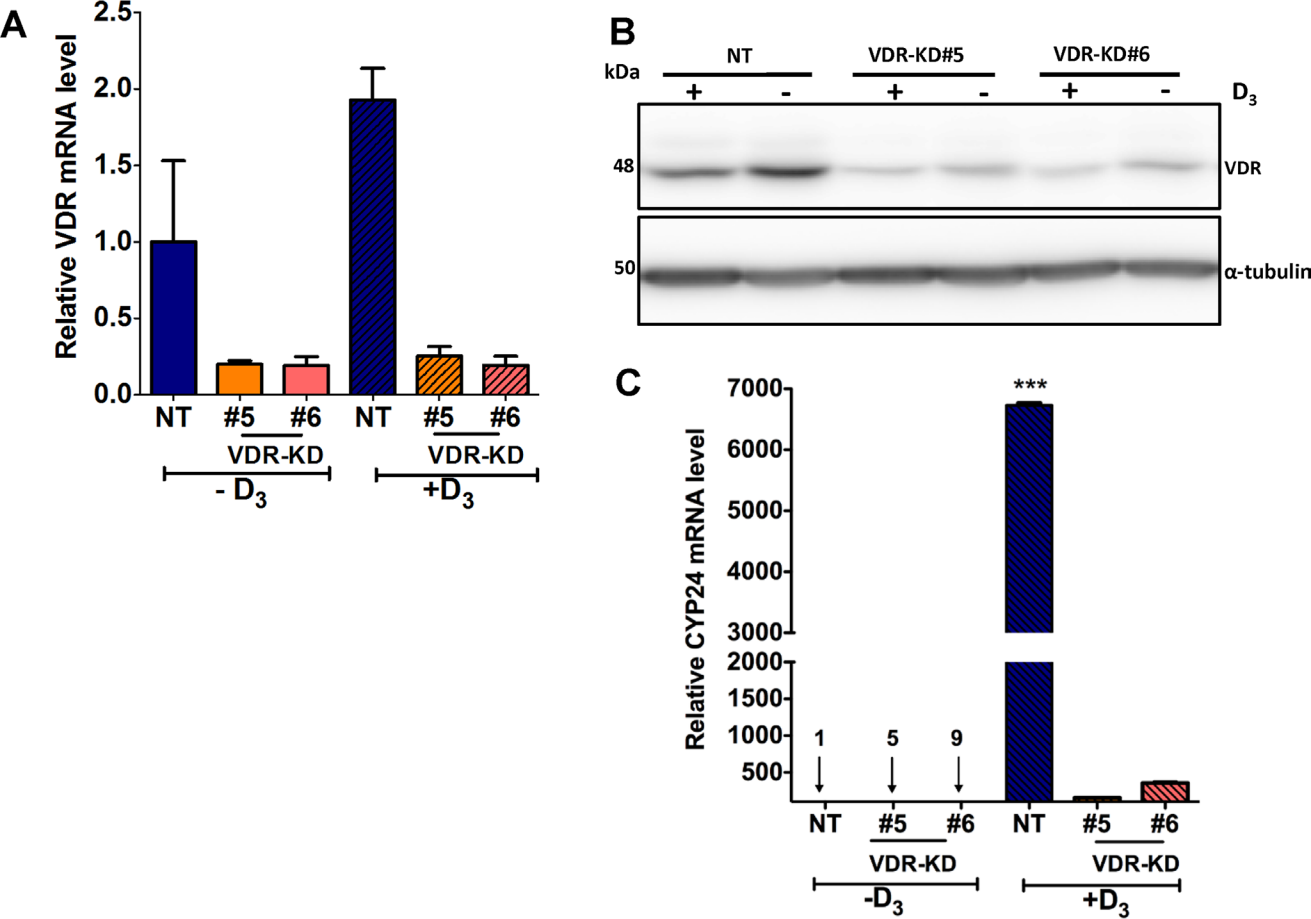
VDR knockdown abrogates vitamin D signaling in MCF-7 cells (**A**) In response treatment with 1,25D_3_ for 24 hours, VDR mRNA was increased by 2-fold in NT cells as compared to vehicle treated cells. In contrast, VDR-KD clones showed marginal response to the 1,25D_3_ treatment. (**B**) After 48-hour treatment with 1,25D_3_, VDR protein levels were significantly increased in NT treated cells. The VDR-KD clones show marginally increased VDR protein levels following treatment. (**C**) After 24-hour treatment of NT cells, a significant induction of *CYP24* mRNA was observed compared to vehicle treated cells. In contrast, *CYP24* mRNA induction was attenuated in VDR-KD clones with 1,25D_3_ treatment. Results are expressed as the mean ± SEM (*n* = 3). ***p < 0.001 NT(−D3) compared to NT (+D3) using one-way ANOVA with Tukey's post test.

Treatment with 10^−8^M 1,25D_3_ for 24 hours increased VDR mRNA and protein expression by NT cells, while the two MCF-7-VDR-KD clones showed only marginal responses to ligand exposure (Figure 2A, 2B). CYP24 is a direct VDR target gene [23, 29] and treatment with 1,25D_3_ induced a robust increase in CYP24 mRNA in NT cells (Figure 2C). In contrast, CYP24 mRNA induction was attenuated in VDR-KD#5 and VDR-KD#6 knockdown clones (Figure 2C), indicating effective disruption of VDR signaling in both clones.

### VDR knockdown reduces MCF-7 cell growth and induces apoptosis in ligand-free culture

**Figure 3:**
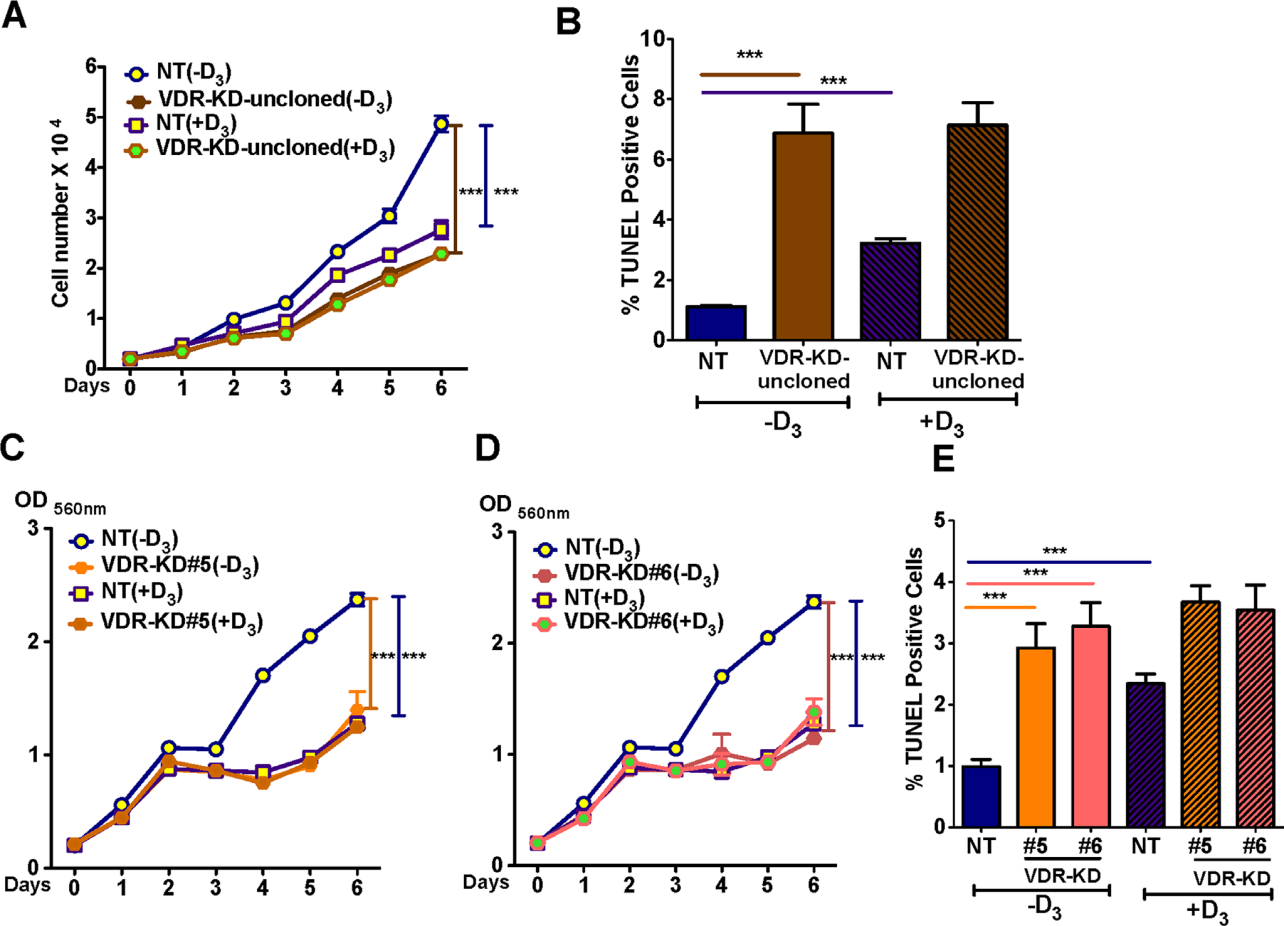
VDR knockdown reduces MCF-7 cell growth and induces apoptosis in a ligand-independent manner (**A**) In ligand-free culture, MCF7-VDR-KD non-clonal cells showed reduced growth by 53% compared to NT cells. Treatment of NT cells with 1,25D_3_ reduced cell growth by 44% compared to untreated cells. (**B**) In ligand free culture, MCF7-VDR-KD uncloned cells showed 6.8-fold increased apoptosis compared to NT cells, as measured by TUNEL assay. In response to 1,25D_3_, NT uncloned cells showed 3-fold increased apoptosis as compared to untreated cells.(**C**) Similar to non-clonal cells, VDR-KD#5 showed 41% growth reduction compared to MCF7-NT cells. Treatment of MCF7-NT cells with 10^−8^ M 1,25D_3_ reduced cell growth by ∼50% compared to untreated MCF7-NT cells. In contrast, the same treatment has no effect on growth of VDR-KD#5 cells. (**D**) Similarly, on day 6, VDR-KD#6 had a decreased growth by 51%, compared to NT in ligand-free culture. The magnitude of growth inhibition in VDR knockdown clones is comparable to the ligand mediated growth inhibition in control cell lines. Treatment of VDR-KD#6 cells with 1,25D_3_ does not reduce cell growth. (**E**) Both VDR-KD clones exhibited a 3-fold increased rate of apoptosis compared to NT cells in ligand-free culture. Upon 1,25D_3_ treatment, NT cell apoptosis increased 1.3-fold while VDR knockdown cells showed slightly (but insignificant) increased apoptosis compared to vehicle treated cells. Results are expressed as the mean ± SEM. For growth assay (*n* = 6) ***p < 0.001 compared to NT (−D3) using 2-way ANOVA with Bonferroni post-test. For TUNEL using 1-way ANOVA with Tukey's post-test.

To test the effect of 10^−8^M 1,25D_3_ on the *in vitro* growth of NT and VDR-KD cells, clonal and non-clonal lines was tested over 6 days, cells were cultured in charcoal-stripped media that lacks 1,25D_3_. The growth rate of MCF-VDR-KD cells prior to clonal selection, when the population was still heterogeneous, was first examined. Compared to vehicle, treatment with 1,25D_3_ significantly reduced the growth of NT cells but not that of VDR-KD cells, consistent with the disruption of VDR signaling. Surprisingly, however, the MCF7-VDR-KD non-clonal cells showed ligand-independent growth inhibitory and pro-apoptotic effects, when compared with MCF7-NT non-clonal cells (Figure 3A, 3B). These results were further confirmed using MCF-7-VDR-clonal lines. Similar to the non-clonal cell line, we found that in the absence of 1,25D_3_ the growth of both VDR-KD clones was significantly decreased compared to NT cells. Remarkably, growth of VDR-KD clones in absence of 1,25D_3_ was similar to that of NT cells in the presence of 1,25D_3_ (Figure 3C, 3D). Furthermore, treatment with 1,25D_3_ increased apoptosis in NT cells. In the absence of ligand, apoptosis in VDR-KD clones was significantly increased compared to NT cells. However, 1,25D_3_ treatment of VDR-KD clones did not increase apoptosis (Figure 3E). These data indicate that loss of the VDR decreases the growth of breast cancer cells and induces apoptosis independently of its ligand, 1,25D_3_.

### VDR knockdown in MCF-7 cells reduces orthotopic tumor growth *in vivo*

**Figure 4:**
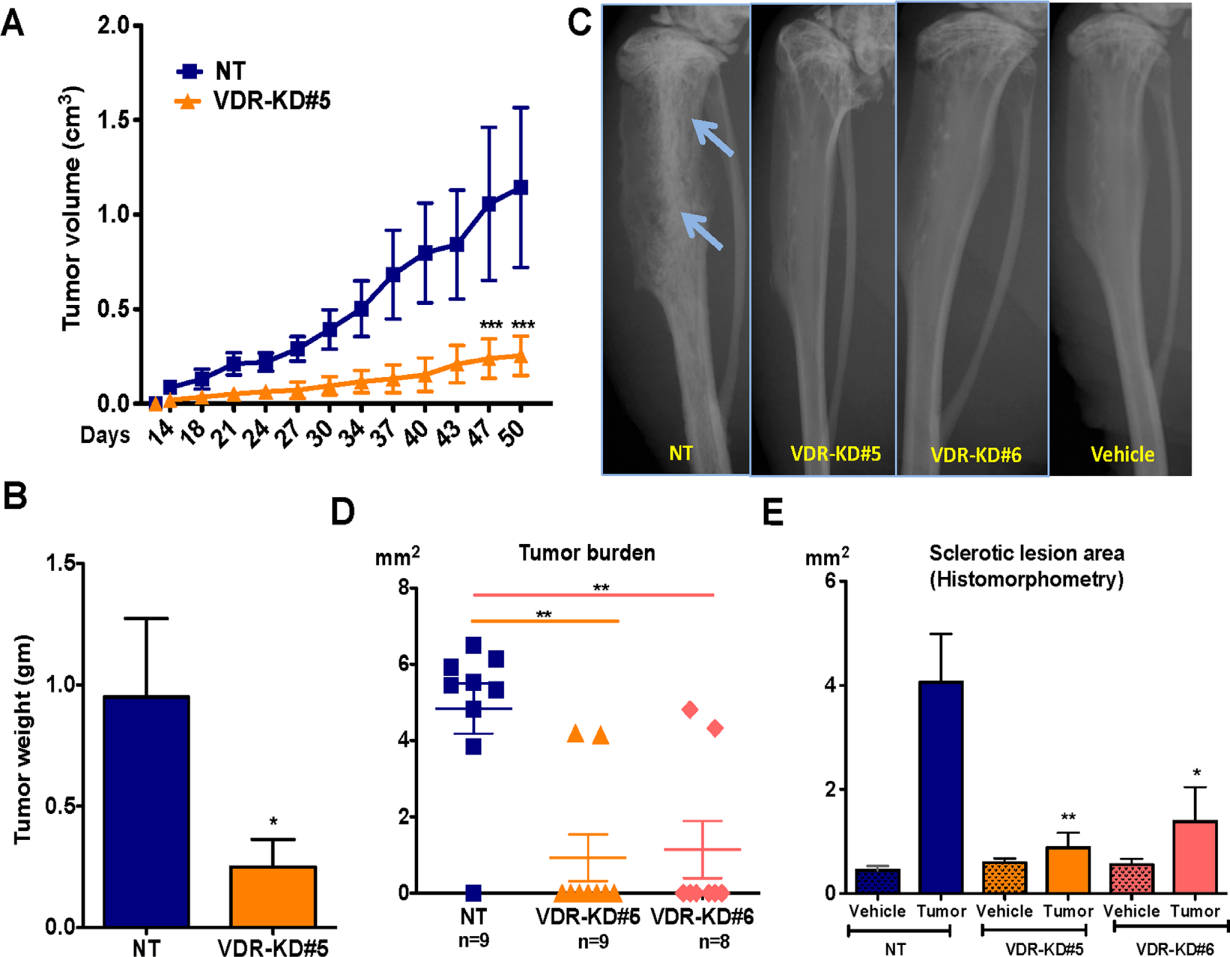
VDR knockdown in MCF-7 cells reduces orthotopic tumor growth and also reduces its capability to form tumor in the bone environments (**A**) Orthotopic tumor growth: Tumors derived from VDR-KD#5 cells grew significantly slower than NT cell-derived tumors. (**B**) Orthotopic tumor weight: At the experiment endpoint (day 50), the tumor mass of mice that received the VDR-KD clone was reduced by 72% compared to MCF7-NT. (**C**) Representative radiographs: Representative images showing osteoblastic lesions as indicated by arrows in tibiae injected with MCF7-NT cells 21 weeks p.i. In contrast, no bone changes can be seen in bones inoculated with VDR-KD clonal cells. (**D**) Quantitative histomorphometry: Of the mice implanted with MCF7-NT, 88% developed tumors as confirmed by the presence of cancer cells in tibiae. In contrast, only 25% of mice implanted with VDR-KD cells developed tumors. (**E**) Bone histomorphometry: Sclerotic lesion area was significantly greater in MCF7-NT injected bones than in tibiae inoculated with VDR-KD cells. Results are expressed as the mean ± SEM: Figure A and B (*n* = 5/group). ***p < 0.001 compared to MCF-N7 NT tumors, using 2-way ANOVA with Bonferroni post-test. Figure C to E (*n* = 8–9/group)., *p < 0.05, **p < 0.01 compared to NT tumor using 1-way ANOVA with Tukey's post-test.

To determine the effects of VDR knockdown in MCF-7 cells on orthotopic cell growth, NT and VDR-KD cells (clone #5) were implanted into the mammary fat pad of vitamin D-replete nude mice. As shown in Figure 4A, VDR knockdown significantly reduced tumor growth compared to MCF-7-NT cells. At endpoint (day 50 post implantation), tumor weight was 72% lower in tumors derived from MCF-7-VDR-KD cells compared to those grown from MCF-7-NT cells (Figure 4B).

### VDR knockdown in MCF-7 cells impairs tumor formation in bone

To determine whether the bone microenvironment affects the growth of VDR-KD cells, NT and VDR-KD clones were inoculated into the proximal tibiae of vitamin D-replete nude mice. Osteosclerotic tumors derived from NT cells became radiographically visible 6 weeks post inoculation (p.i.), and continued to develop until study completion at 21 weeks p.i. In contrast, no obvious X-ray changes were identified in the two VDR-KD groups throughout the duration of the experiment (Figure 4C). Quantitative histomorphometry demonstrated that in the NT-inoculated group, 8 out of 9 mice formed tumors. In contrast, only 2 out of 9 mice inoculated with VDR-KD cells developed histologically identifiable tumors, while in the remaining 7 mice no tumor was present (Figure 4D). Tumor area was 4.7-fold lower in tibiae inoculated with VDR-KD#5 compared to NT cells, and similar differences were observed with VDR-KD#6 cells (Figure 4D). Trabecular bone volume (sclerotic lesion area assessed histomorphometrically) was increased in all groups compared to vehicle-inoculated tibiae. However, compared to NT controls, the amount of trabecular bone volume was significantly lower in tibiae inoculated with VDR-KD cells (Figure 4E).

**Figure 5:**
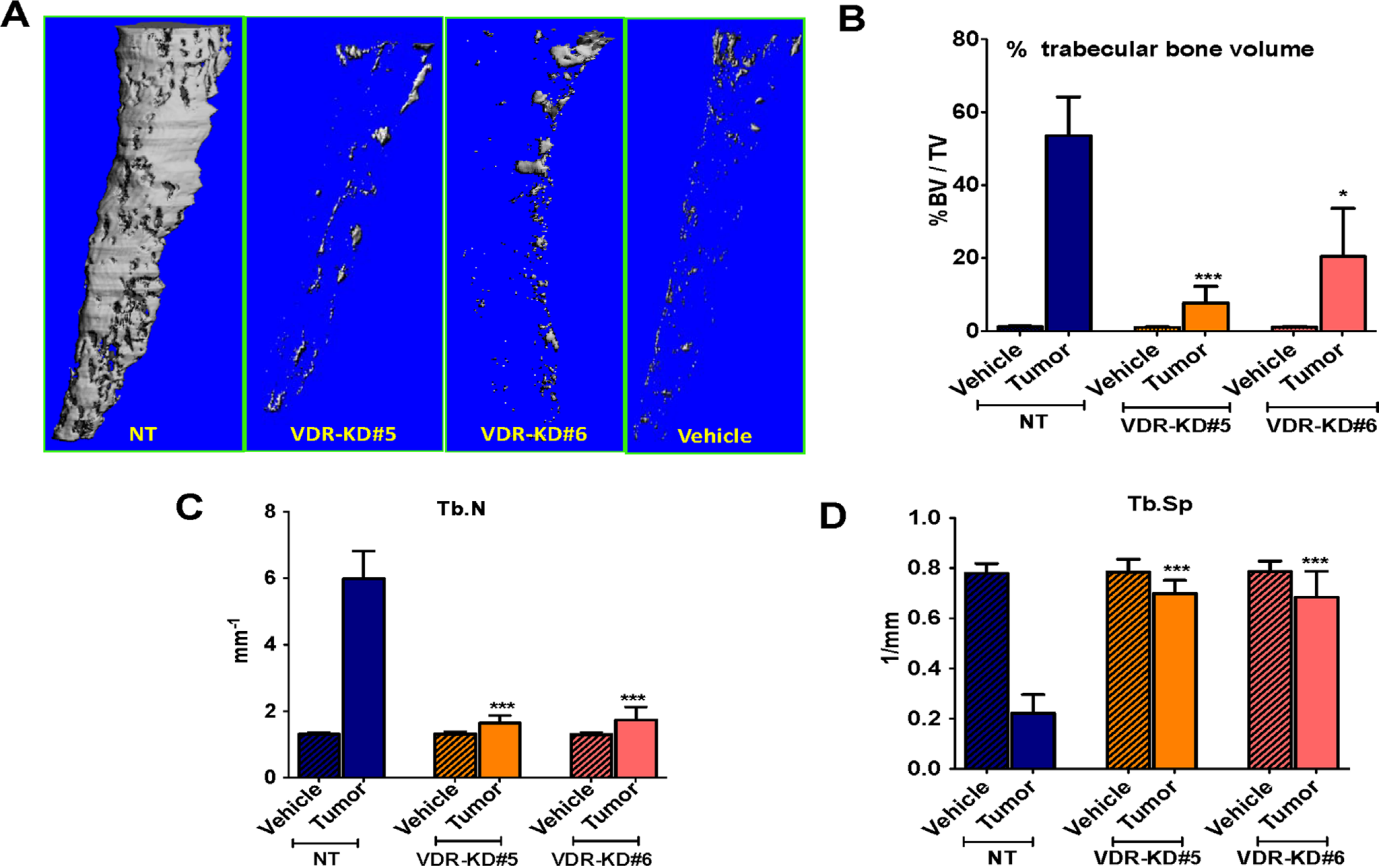
VDR knockdown in MCF-7 cells decreased osteosclerotic bone formation in bones of nude mice Bone micro CT analysis was performed only for trabecular bone by excluding cortical bone (**A**) Bone microCT: Representative images showing tumor-mediated trabecular bone formation in tibiae injected with MCF7-NT cells 21 weeks p.i. In contrast, negligible amounts of trabecular bone formation seen in bones inoculated with VDR-KD clonal cells. (**B**–**D**) Quantitative analysis of micro-CT scans: Increased trabecular bone volume (BV/TV %) was observed in tibiae injected with NT cells compared to contralateral tibiae and VDR-KD inoculated tibiae. Trabecular number in the tibiae inoculated with NT cells was significantly higher compared to vehicle control. Tibiae injected with VDR knockdown cells showed significantly higher trabecular number compared to vehicle. Similarly, trabecular separation was decreased by 72% in tibiae with NT cells, compared to vehicle inoculated tibiae. Trabecular separation in VDR-KD inoculated tibia was reduced up to 13% compared to vehicle-inoculated tibiae. Results are expressed as the mean ± SEM. *p < 0.05, ***p < 0.001 compared to NT tumor using 1-way ANOVA with Tukey's post-test.

MicroCT analysis revealed that trabecular bone volume of tibiae inoculated with NT cells was increased by 44-fold compared to vehicle-injected bones. In contrast, trabecular bone volume in tibiae inoculated with VDR-KD#5 and VDR-KD#6 cells increased by only 8 and 18-fold, respectively, compared to vehicle-injected controls, again significantly less than bones inoculated with NT cells (Figure 5A, 5B). Trabecular number (Tb.N) in the tibiae inoculated with NT was significantly higher than in contralateral tibiae as well as the VDR-KD inoculated tibiae (Figure 5C). Similarly, trabecular separation (Tb.Sp) in VDR-KD containing bones was 2-fold higher than in NT bearing bones (Figure 5D).

Trabecular number in the tibiae inoculated with NT cells was significantly higher (+456%) compared to vehicle control. Tibiae injected with VDR knockdown cells showed an increase in trabecular number of up to 141% compared to vehicle (Figure 5C). Similarly, trabecular separation was decreased by 72% in tibiae with NT cells, compared to vehicle inoculated tibiae. Trabecular separation in VDR-KD inoculated tibia was reduced up to 13% compared to vehicle-inoculated tibiae (Figure 5D).

Collectively, these data indicate that VDR knockdown negatively affects the capability of MCF-7 cancer cells to form tumors and induce osteosclerosis in bone following direct intra-tibial inoculation in mice.

### Stably expressed mutant VDR (mutVDR) accumulates in the cytoplasm

As shown above, knockdown of the VDR in MCF-7 breast cancer cells reduced growth in ligand-free conditions, suggesting that the VDR functions independently of 1,25D_3_ binding to promote cell growth *in vitro*. To further elucidate the function of the VDR in breast cancer cells and the underlying molecular events. Therefore, we next tested the hypothesis that the cytoplasmic VDR controls breast cancer cell growth.

**Figure 6:**
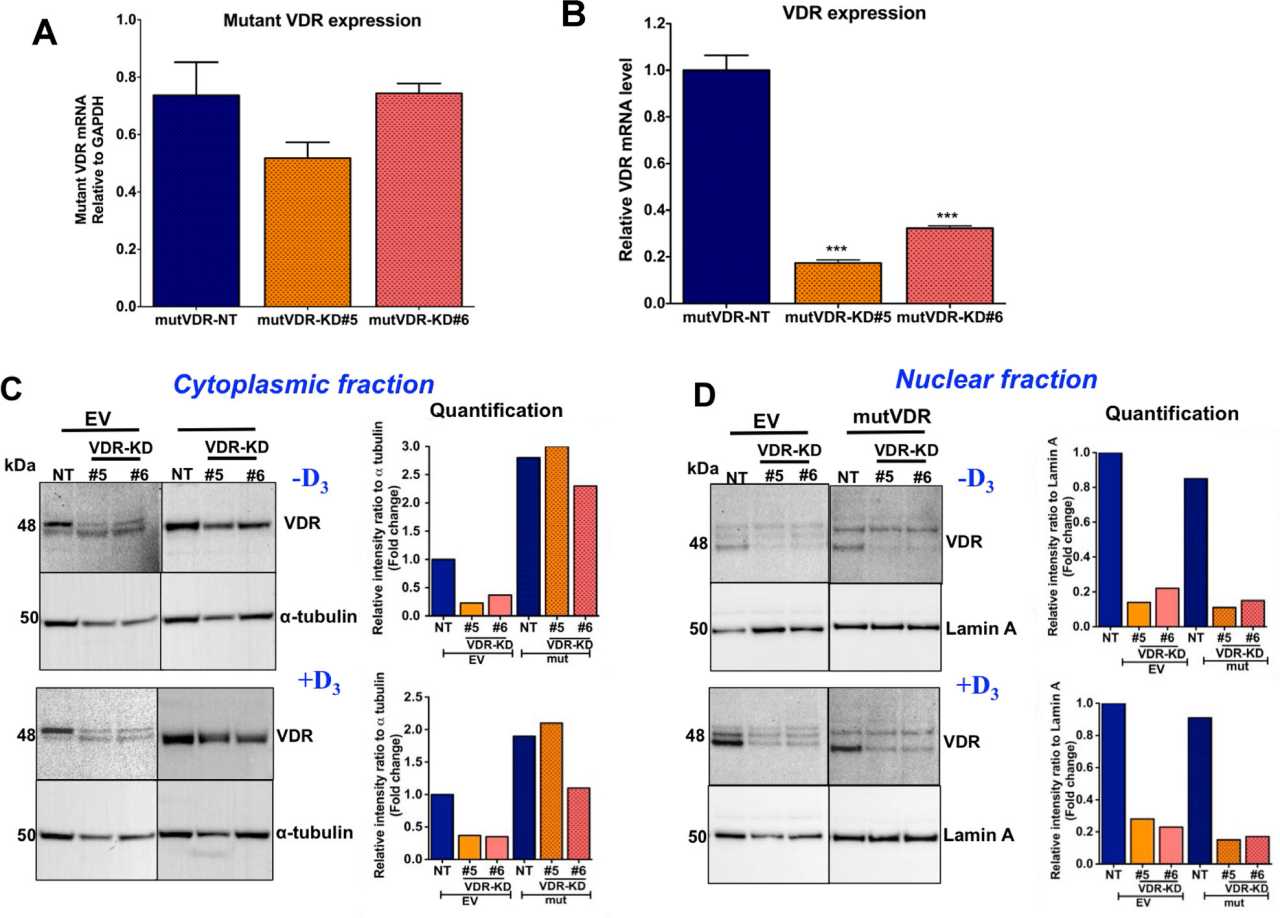
Stable expression of nuclear localization mutant VDR (mutVDR) in VDR knockdown clones (**A**) After stable transfection of mutVDR into VDR knockdown cells the level of mutVDR mRNA in VDR knockdown clones was comparable to MCF7-NT cells using mutant VDR specific primers. (**B**) Expression of mutVDR in VDR-KD#5 and VDR-KD#6 does not change its shRNA knockdown effects. (**C**) Western blot of cytoplasmic fractions and its quantification. After stable transfection of the mutVDR and empty vector (EV) into VDR knockdown cells, Western blots demonstrated a ∼3-fold increase in mutVDR protein in the cytoplasmic fraction of VDR-KD clones compared to EV-transfected NT cells in the absence of vitamin D. (**D**) Western blot of nuclear fractions and its quantification. Despite stable transfection of mutVDR and EV into VDR-KD cells, the mutVDR marginally translocated into the nucleus following treatment with 10^−8^M 1,25D_3_ for 48 hours. In contrast, the intact VDR considerably translocated to the nucleus in EV transfected control cells in response to treatment with 1,25D_3_. Results are expressed as the mean ± SEM. ***p < 0.001 compared to mutVDR-NT using 1-way ANOVA with Tukey's post-test.

To explore this hypothesis, we introduced a mutant VDR construct which, due to a mutation in the nuclear localization signal in the VDR gene, is unable to translocate to the nucleus and thus accumulates in the cytoplasm [30]. Importantly, the ligand-binding domain of the mutant VDR is not modified and thus is available to bind 1,25D_3_ [31]. As shown in Figure 6A, mRNA expression levels of the mutVDR (measured using specific primers that detect mutVDR expression only) were similar in mutVDR-NT and mutVDR-VDR-KD cells. To confirm that expression of the mutant VDR in VDR-KD cells did not affect the efficiency of knockdown of endogenous VDR, we determined the levels of endogenous VDR mRNA using specific primers. As shown in Figure 6B, VDR-KD cells expressing the mutant VDR continued to show a knockdown of the endogenous VDR, confirming that the efficiency of shRNA knockdown of VDR is not reduced with concurrent mutVDR expression in these cells. VDR Western blots demonstrated ∼3-fold increase in VDR protein levels in the cytoplasmic fraction of mutVDR-VDR-KD cells compared to empty vector (EV)-NT cells (Figure 6C). Upon treatment with 1,25D_3_, VDR is normally detectable in the nuclear fraction, as is evident in EV-transfected cells. In contrast, mutVDR is much less responsive to 1,25D_3_–induced nuclear translocation (Figure 6D). Of note, complete abrogation of nuclear VDR translocation was not demonstrated with this mutation, which may have resulted in a marginal increase in nuclear VDR signals in mutVDR expressing VDR-KD cells.

### Cytoplasmic VDR positively controls MCF-7 breast cancer cell growth in the absence of vitamin D

**Figure 7:**
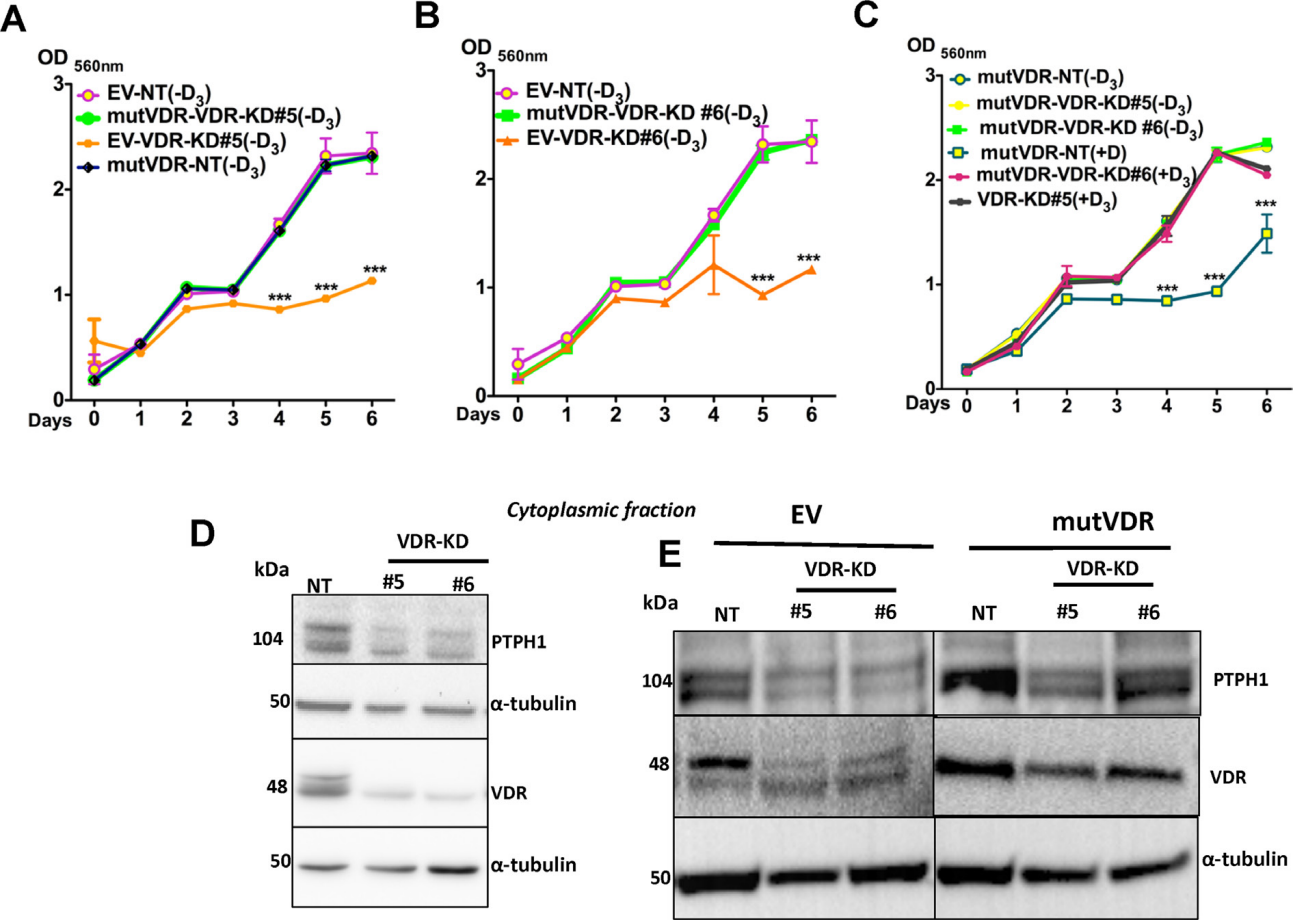
Expression of mutVDR into the VDR knockdown cells restores their growth (**A**–**B**) Similar to VDR-KD cells, in ligand-free culture the EV-VDR-KD clones were slow in growth compared to EV-NT. Stable expression of the mutVDR in VDR-KD clones rescued the growth phenotype of VDR knockdown cells. mutVDR expression in both knockdown clones resulted in similar increases in growth rate. This was comparable to the growth rate of EV-NT cells. (**C**) When mutVDR -VDR-KD clones were treated with 1,25D_3_, they were unresponsive to treatment, thus cell growth remained the same until day 5. A slight reduction in growth was observed on day 6. (**D**) Western blots of the cytoplasmic fraction showed that compared to NT, PTPH1 protein levels were decreased by 50% in VDR-KD#5 and 40% in VDR-KD#6 (**E**) After mutVDR expression, PTPH1 protein was increased by 2 fold in cytoplasm of mutVDR -VDR-KD clones compared to their respective EV controls. Results are expressed as the mean ± SEM (*n* = 6). ***p < 0.001 compared to EV-NT (−D3), mutVDR-VDR-KD#5 (−D3), mutVDR-VDR-KD#6 (−D3), mutVDR-NT(−D3), mutVDR-VDR-KD#5 (+D3), mutVDR-VDR-KD#6 (+D3) using 2-way ANOVA with Bonferroni post-test.

We next compared the growth of EV and mutVDR expressing cells *in vitro* in the presence and absence of 1,25D_3_. The growth of the EV-transfected cells (including EV-NT and EV-VDR-KD) was comparable to that of non-transfected cells. In the absence of ligand, the growth of mutVDR -transfected NT cells was similar to EV transfected NT cells (Figure 7A). As expected, the EV-VDR-KD clones demonstrated reduced growth in the absence of ligand compared to EV-NT cells. However, stable expression of the mutVDR in VDR-KD clones increased their growth rate to that of EV-NT cells (Figure 7A, 7B). These results indicate that the VDR is involved in the regulation of MCF-7 cell growth through its cytoplasmic accumulation. When mutVDR-VDR-KD cells were treated with 1,25D_3_, they were unresponsive to the treatment and there was no suppression of cell growth until day 5 (Figure 7C).

Protein tyrosine kinases and protein tyrosine phosphatases (PTPs) play a critical role in malignant transformation through their tightly regulated activity on tyrosine phosphorylation. Protein tyrosine phosphatase 1 (PTPH1) has been identified as a specific phosphatase for p38γ mitogen-activated protein kinase (MAPK) that shows oncogenic activity in cooperation with MAPK through direct binding. In human breast cancer cells, PTPH1 regulates cancer cell growth by its stimulatory effect on VDR via its binding to VDR in the cytoplasm [32, 33]. To confirm the ligand-independent cytoplasmic function of VDR, we measured PTPH1 protein levels in NT and VDR-KD cells. In ligand-free conditions, PTPH1 protein in cytoplasmic extracts was decreased by 50% in VDR-KD#5 and by 40% in VDR-KD#6 clones compared to NT cells (Figure 7D). When the mutVDR was expressed in VDR-KD cells (mutVDR-VDR-KD), PTPH1 protein levels were increased by 2-fold in mutVDR-VDR-KD clones relative to their respective EV controls (Figure 7E). These results support the hypothesis that cytoplasmic VDR promotes MCF-7 breast cancer cell growth possibly through its action on PTPH1.

## DISCUSSION

By using a stable VDR knockdown approach *in vitro* and *in vivo* in mouse models, our study demonstrates that the cytoplasmic VDR plays an important role in breast cancer cell growth. This novel ligand-independent function of the VDR to promote cancer cell growth contrasts with its canonical ligand-dependent inhibitory nuclear actions on cell growth in the presence of vitamin D.

The MCF-7 breast cancer cell line expresses an abundance of endogenous VDR, which makes it difficult to achieve efficient VDR knockdown. Therefore, we performed single cell clonal selection to isolate two clones with marked reduction in VDR expression (∼ −85%) using two controls, PA and NT. The knockdown of VDR efficiently blocked VDR-mediated signaling as shown by reduced expression of the VDR primary response gene *CYP24* in the presence of vitamin D.

Consistent with previous reports [23, 26, 34], we showed that vitamin D treatment inhibited MCF-7 cell growth. Based on previous studies that vitamin D deficiency promotes breast cancer growth in bone [26, 27, 35] as well as the known anti-proliferative effects of vitamin D on breast cancer cells, we originally hypothesized that knockdown of the VDR in breast cancer cells would promote tumor cell growth. As expected, knockdown of the VDR in MCF-7 cells rendered the cells insensitive to vitamin D. However, in contrast to our hypothesis, we were surprised to find that VDR knockdown alone, in the absence of vitamin D, impaired cell growth. These results suggest that the VDR may have the hitherto unknown ability to stimulate cell growth independent of ligand binding. Experiments measuring the growth of two VDR-KD clones and VDR-KD uncloned cells using two different assays (MTT and direct cell count) provided the same results, indicating that the growth inhibitory phenotype of VDR-knockdown cells is not due to off-target effects or an artifact in the MCF-7-KD cells.

Our *in vivo* studies were performed in vitamin D-replete mice. Of note, the VDR-KD cells were consistently non-responsive to vitamin D treatment in the *in vitro* experiments, which provides evidence that the growth suppression seen in VDR-KD cells *in vivo* is independent of vitamin D. The bone microenvironment provides a fertile soil for breast cancer cells, and bone is a preferred target organ for breast cancer metastasis [7, 36, 37]. MCF-7 cells have previously been reported to form mixed osteolytic and osteoblastic bone lesions in which both osteoclasts and osteoblasts act to support tumor growth [36, 38, 39]. Our *in vivo* experiments indicate that ablation of the VDR in MCF-7 cells strongly reduces tumor formation within the bone environment, since about 87% of the mice failed to develop histologically visible tumors or tumor-related changes in the bone. In contrast, visible osteoblastic tumors were seen in 8 out of 9 tibiae inoculated with NT cells. The tumors seen in the two mice that did develop visible lesions following injection of VDR-KD clones were comparable in size and structure to those formed in mice injected with NT. These tibiae also showed a radiographic phenotype equivalent to that seen in the MCF7-NT group. One reason for the apparent impairment in tumor development with VDR-KD cells could be that the time required for inoculated cells to commence rapid proliferation and form tumours in VDR-KD cells is longer than that for the control cells. Alternatively, it could represent a reduced ability of VDR-KD cells to survive inoculation and introduction to a bone environment.

As the ligand-independent function of the VDR has not been widely studied, little information is available on the ligand-independent properties of the VDR. In skin cells, the unliganded VDR is required for keratinocyte stem cell function through interaction with the Wnt-β-catenin signaling pathway [40, 41]. In breast cancer, a single *in vitro* study has identified that PTPH1 positively regulates breast cancer growth by its stimulatory effect on VDR protein expression [32]. VDR localization and its specific effects are an emerging field of research and the findings we reported in this study contribute to our understanding of the phenotypic effects of cytoplasmic VDR on cancer cells. Furthermore, it has been reported that in the absence of its ligand, the VDR is distributed between the cytoplasm and the nucleus [30, 42]. VDR localization and its dependence on ligand binding are not absolute, though, as shown by us and others [24, 26, 27, 29], ligand binding is associated with enhanced shuttling of the VDR from the cytoplasm to the nucleus. However, recent evidence suggests that shuttling of the unliganded VDR to the nucleus produces basal transcriptional activity via binding to co-repressors or co-activators [43–47]. Global chromatin immunoprecipitation (ChIP) analyses have also indicated that a significant number of DNA-binding sites are occupied by the VDR in the absence of its cognate ligand [48]. However, the role of cytoplasmic VDR in the absence of ligand is not well studied. Our VDR knockdown results suggest a different role than expected and have led us to propose that the cytoplasmic VDR could be a factor contributing to the regulation of breast cancer cell proliferation. To investigate this further, we successfully created conditions that induced accumulation of VDR in the cell cytoplasm using stable expression of a mutant form of VDR.

Stable expression of mutVDR increased the growth of VDR knockdown cells to that of unmodified NT cells. This experiment demonstrated that in absence of its ligand, the cytoplasmic VDR promotes cell growth, a function that is in stark contrast to the inhibitory effects of the liganded VDR following nuclear translocation. Using Western immunoblotting, we have detected the presence of PTPH1 protein in cytosolic extracts of NT cells and its absence in VDR-KD cells. When the VDR was artificially accumulated in the cytoplasm of MCF7-KD cells using the mutant VDR, PTPH1 also increased corresponding to increased cytoplasmic VDR expression. These results support the concept that the cytoplasmic VDR controls MCF-7 cell growth. Altered PTPH1 levels with respect to VDR localization may be the responsible factor mediating the actions of unliganded cytoplasmic VDR but the detailed molecular interactions between their cytoplasmic association and cellular response remain to be elucidated.

Vitamin D deficiency is common in women with breast cancer at advanced disease stages [8–10]. However, the relationship between vitamin D status and clinical outcomes remain controversial. Numerous studies have linked low serum concentrations of 25-hydroxyvitamin D with increased cancer incidence and more aggressive tumor behavior, including increased bone metastasis and decreased survival [8, 10, 49]. However, most of these reports are epidemiological or clinical association studies, which cannot establish cause and effect, nor do they provide plausible mechanistic concepts. It is therefore not surprising that other reports are inconsistent with the relationship suggested by clinical and epidemiological studies [50, 51]. As the VDR is the central modulator of the autocrine/paracrine response to vitamin D, some of the inconsistencies in the above quoted clinical studies regarding the correlation between vitamin D status and breast cancer might be due to differences in VDR status [18, 52]. Our mostly observational study indicates that apart from vitamin D levels, VDR status and its compartmental distribution may contribute to regulating breast cancer cell growth. The use of a single model cell line is a limitation of this study and it is currently unknown if cytoplasmic VDR accumulation causes similar effects in other cancer cell lines. Detailed investigation into mechanisms behind ligand-independent cytoplasmic activity of VDR under conditions of vitamin D deficiency would have clinical applicability in deciding the treatment strategies in patients with breast cancer. Measuring nuclear and cytoplasmic VDR expression in human tumors at different stages of progression and correlating these results with the patient's vitamin D status would help to clarify whether cytoplasmic accumulation of VDR might be relevant in regulating tumor growth in patients. A prospective clinical trial would be required to determine such a relationship, and this could help clarify the clinical discrepancies reported with regard to vitamin D and tumor outcomes [50].

In conclusion, this study demonstrates that cytoplasmic VDR can control MCF-7 breast cancer cell growth independent of vitamin D. Further investigations into the underlying mechanisms may deepen our understanding of the role of the VDR in cancer cell behavior and thus may open new therapeutic approaches for patients with metastatic breast cancer.

## MATERIALS AND METHODS

### Cell line

The human breast cancer cell line MCF-7 was selected for these studies as it expresses the VDR at relatively high levels and is responsive to 1,25D_3_ [24]. MCF-7 cells were obtained from Garvan's Cancer Group which is now known as Kinghorn Cancer Centre. We have authenticated these cells as human by probing RNA with human specific probes for housekeeping genes and confirmed their expected phenotype of high estrogen dependence and high sensitivity to growth inhibition by 1,25D_3_ treatment, and by their induction of mixed osteogenic/osteoblastic lesions when implanted in tibiae. MCF-7 cells were maintained in RPMI medium supplemented with 10% fetal bovine serum (FBS), 1% penicillin-streptomycin solution and 200 μg/mL insulin (Sigma, MO, USA) in a 37°C humidified atmosphere with 5% CO_2_. All the experiments used cells of ≤ 5 passages. Cells were routinely tested for the absence of mycoplasma contamination using mycoplasma GoTaq M5005 detection kit (Promega Corporation, WI, USA).

### Stable shRNA knockdown and preparation of stable clones

Parental MCF-7 cells (PA) were transduced with a lentivirus encoding shRNA against the VDR (shVDR, TRCN000019506, MISSION shRNA, Sigma) (VDR-KD). Cells transduced with a non-target shRNA control vector (shNT, SHC002V, Sigma) were used as controls (NT). Two VDR-shRNA constructs were tested and the one with best knockdown efficiency was used for VDR knockdown and subsequent clonal selection. VDR-KD and NT cells were selected from heterogeneous pools using 2 μg/mL puromycin (Sigma). Clonal selection for both NT and VDR-KD was carried out using limiting dilution in the presence of puromycin in a 96-well plate format. Using this method, 30 NT and 27 VDR-KD clones were prepared, maintained and screened at mRNA and protein levels to identify clones with high VDR knockdown efficiency. From these, one NT and two VDR-KD (#5 and #6) clones were selected for further *in vitro* and *in vivo* experiments. The selection of two VDR knockdown clones reduced the possibility of clonal as well as off-target effects. Throughout this study both the knockdown clones showed a similar phenotype. Initially, we used both PA and NT as controls, however as they each showed a similar phenotype we continued all experiments using NT as a control since it had been generated in parallel with the VDR-KD clones.

### Nuclear localization signal (NLS) mutant VDR (mutVDR) overexpression

The NT and VDR-KD MCF-7 cells were transfected with a mutated form of the VDR (mutVDR), which due to a mutation in the nuclear localization (NLS) signal is unable to translocate to the nucleus (kindly provided by Prof Chen, Medical College of Wisconsin, USA). The mutVDR was originally generated by Prufer *et al*. through point mutations of the K53Q, R54G and K55E basic amino acids in the NLS-I of VDR and cloned into a V5-tagged pcDNA3.1 vector [30]. A pcDNA3.1 empty vector (EV) was used as a control. MCF-7 cells (NT and VDR-KD clones) were plated in 6-well plates at 3 × 10^5^ cells/well and transfected using Lipofectamine^TM^ (10 μg DNA per well) [53]. From the next day forward, cells were selected using the G418 antibiotic (1 mg/mL) for 3–5 days. After selection, mutVDR -expression was confirmed using real time-PCR and western blotting. For the purpose of this study, the cells resulting from this transfection will be referred to as mutVDR -NT, mutVDR -VDR-KD, EV-NT and EV-VDR-KD.

### Quantitative RT-PCR

For gene expression studies, total RNA was isolated using GenElute RNA miniprep kits (Sigma) or Nucleospin RNA II kits (Macherey-Nagel GmbH & Co. KG) from the cells treated with 10^−8^M 1,25D_3_ (Biomole, ENZO life sciences, NY, USA) or its vehicle (ethanol) for the times indicated under serum-free conditions (0.1% BSA containing RPMI media supplemented with 1% penicillin streptomycin solution and 200μg/mL insulin). Total RNA (500 ng/sample) was reverse-transcribed using Superscript III (Life Technologies, CA, USA) after oligo(dT) primers (Promega Corp, WI, USA). Real-time PCR was performed using Hot start-IT SYBR green Q-PCR master mix (Affymetrix, Inc. USB products, CA, USA) with Bio-Rad CFX96 Thermal Cycler (Bio-Rad, CA, USA), or alternately, using iQSYBR-Green SuperMix (Bio-Rad) with Bio-Rad thermal cycler (Bio-Rad). Primers were optimized for RT-PCR target gene expression, which was normalized against the housekeeping gene, GAPDH. mRNA levels for gene expression of human GADPH, VDR, CYP24A1, and mutant VDR were assessed by real-time RT-PCR using species-specific primers. Primer sequences were: hGAPDH (Forward 5′-TATGACAACGAATTTGGCTACAG-3′; Reverse 5′-GATGGTACATGACAAGGTGC-3′); hVDR (Forward 5′-ACCTGGACAACAAGAGCGA-3′; Reverse 5′-CTCCTTCCTTCTCCTTCTGATG-3′); hCYP24A1 (Forward 5′-GCATCTTCCATTT GGCGT-3′; Reverse 5′-AATACCACCATCTGAGGCGT-3′); hmutantVDR (Forward 5′-CCACCTGCTCTATGCCAAG-3′; Reverse 5′-GAAAGGACAGTGGGAGTG-3′).

### Cell fractionation and Western blot analysis

NT and VDR-KD cells or their mutVDR or EV transfected counterparts were treated with 10^−8^M 1,25D_3_ or vehicle (0.1% ethanol) in RPMI medium supplemented with 2% charcoal stripped, heat-inactivated FBS and 200 μg/mL insulin. In our hands, untreated FBS contains 1,25D_3_ at a concentration of 360pM when measured by specific RIA (Diasorin, USA). In contrast, 1,25D_3_ concentrations were undetectable in media supplemented with 2% charcoal stripped heat-inactivated FBS. Moreover, when MCF-7 cells were cultured in serum-free media or in media supplemented with 2% charcoal stripped heat-inactivated FBS, induction of CYP24 expression was not observed. We therefore considered culture media supplemented with 2% charcoal stripped heat-inactivated serum, 1% penicillin streptomycin solution and 200 μg/mL insulin to be free of 1,25D_3_ and refer to it as ligand-free media.

Media was changed every 24 hours. After 48 hours, whole cell lysates were prepared using RIPA buffer supplemented with protease inhibitor cocktail (Roche Pharmaceutical Co., Germany). Total protein concentration was determined using a Bradford assay (BioRad, CA, USA). Samples were loaded on 8% polyacrylamide gels and electrophoresis was performed using a Mini Trans-Blot cell (BioRad). Proteins were transferred onto a Hybond™-P membrane (GE Healthcare, WI, USA) using a Mini-PROTEAN Cell transfer system (Bio-Rad). Alternatively, cell fractions were prepared using NE-PER Nuclear and Cytoplasmic Extraction reagent kits (Thermo Scientific, IL, USA) followed by protein estimation using a BCA protein assay (Thermo Scientific, IL, USA). Samples were loaded on Bolt, 4–12% mini gels, separated and transferred using the Bolt electrophoresis system and reagents (Invitrogen, CA, USA).

Membranes were blocked in TBS-T-5% milk for 1 h, incubated overnight with the primary antibody and for 1 h with the secondary antibody. Antibody detection was performed using Immobilon^™^ Western Chemiluminescent HRP Substrate (Millipore, MA, USA) according to the manufacturer's directions, and signal was detected either on Image Quant LAS4000 (GE healthcare) or BioRad Image gel system (BioRad). Primary antibodies included anti-VDR (Clone 9A7γ.E10.E4, Neo Markers, CA, USA), anti-PTPH1 (Santa Cruz Biotechnology, CA, USA), anti-α-tubulin (Sigma), anti-Lamin-A (AbCam, MA, USA). Secondary antibodies included anti-Rat (R&D system, MN, USA), anti-goat (Santa Cruz Biotechnology) Anti-mouse IgG, and anti-rabbit IgG (Sigma). A Western blot of VDR from cell extracts showed several bands, consistent with previously published studies using an anti-VDR antibody clone similar to that employed in our study [54–56].

### Cell growth assay

Proliferation of NT and VDR-KD cells, or their mutVDR or EV transfected counterparts, was assessed either by MTT assay or using direct cell counting. Cells were seeded in 2% charcoal stripped FBS (see above) containing RPMI media and were treated with either 10^−8^M 1,25D_3_ or vehicle (0.1% ethanol) and media were replaced every 24hrs. For the MTT assay, MTT reagent (5mg/mL) (Calbiochem, CA, USA) was added to each well. After incubation for 4hrs at 37°C, cells were lysed using a PBS solution with SDS (10%) and HCl (0.01N), and the plate was incubated at 37°C for an additional 16 hrs. Absorbance was measured at 570 nm using an OPTIMA (BMG lab tech, VI, USA) plate reader. Alternatively, treated cells were counted daily by trypan blue exclusion after trypsinizing until they reached 100% confluence on day four. Experiments were repeated 3 times in independent settings to ensure validity of the results.

### Cell apoptosis assay

NT and VDR-KD cells were cultured in medium containing 2% charcoal stripped FBS and treated with either 0.1% ethanol or 10^−8^M 1,25D_3_ for 6 days. Culture media was replaced every 24 hrs. Apoptosis was measured by TUNEL assay using the *in situ* cell death detection kit, POD (Roche Pharmaceutical Co., Germany). TUNEL-positive and total cells were counted in 3–5 random fields/well at 400× magnification.

### Animal experiments

Four-week-old female athymic nude mice with body weight of 18–20 grams were purchased from Harlan (Indianapolis, IN, USA). Animal experiments were performed at Indiana University School of Medicine, IN, USA following approval by Indiana University Institutional Animal Care and Use Committee.

### Mammary fat pad tumor model

Twenty 4-week-old female nude mice were randomized into 2 groups (*n* = 5/group). Both groups of mice were implanted with sustained release pellets containing 0.72 mg of 17Δ estradiol (E_2_) (Innovative Research of America, FL, USA) subcutaneously on the lateral side of the neck, followed by subcutaneous implantation of NT and VDR-KD#5 cells [54]. Cells were suspended in cold Matrigel/PBS (1:1) at a concentration of 2 × 10^7^ cells/mL. 100 μL of the suspension (2 × 10^6^ cells) was implanted into the fourth mammary fat pad using a 27-gauge needle [26]. Starting from day 14, tumor dimensions were measured every three days using a precision calliper and tumor volume was calculated using the formula (length × width^2^)/2. Tumor weight was measured at the termination of the study (day 50).

### Intratibial xenograft model

Fifty-two 4-week-old female nude mice were divided into three groups (*n* = 13/group): NT, VDR-KD#5, VDR-KD#6. Under general anaesthesia with ketamine/xylazine 75/10 mg/kg i.p., 2 × 10^5^ cells in 20 μL of cell suspension (NT and VDR-KD clonal lines) were slowly injected through the knee joint into the tibial plateau of the left tibia using a Hamilton syringe [53, 57]. Contralateral tibiae were injected similarly with vehicle alone (PBS). As MCF-7 cells produce predominantly osteoblastic lesions, tumor growth was monitored every three weeks by digital radiography using a Kubtec Xpert80 (Kubtec digital X-ray, Milford, CT, USA).

### Micro-computed tomography (Micro-CT)

MicroCT analyses were performed using a VivaCT 40 scanner (Scanco Medical, Bassersdorf, Switzerland). Mice were positioned on the microCT bed under continuous isoflurane anesthesia and oxygen. After defining the region of interest, the proximal tibia was scanned at high resolution with 10 μm voxel size and 200 ms integration time. For the trabecular bone analysis, trabecular contours were drawn beginning 300 μm below the growth plate and extending for 7.6 mm -9.5 mm. The same number of slices were analysed in both legs. Parameters measured included: trabecular bone volume per total volume (BV/TV, %), trabecular number (Tb.N, mm^−1^) and trabecular separation (Tb.Sp, mm). All values were compared with the contralateral vehicle injected tibia.

### Bone histology and histomorphometry

Tibiae collected from 6–8 mice/group were used for paraffin embedding and 5–6 mice/group for plastic embedding. Tibiae from mice inoculated with tumor/sham intratibially were collected after euthanasia and fixed in 10% neutral buffered formalin for 48 h, followed by decalcification in 10% EDTA for 3 weeks. After embedding in paraffin, longitudinal mid-sagittal sections of 3.5 μm in thickness were cut and stained with haematoxylin & eosin (MUTVDR&E). Plastic embedded specimens were cut using an automated microtome (Microm HM 360, Thermo Scientific). Longitudinal mid-sagittal sections of 4.5μm in thickness were placed on slides coated with Haupt's solution and incubated at 37°C overnight. Sections were then stained with Von Kossa MacNeal's Tetrachrome to distinguish newly formed osteoid from mineralized bone. Both paraffin and plastic embedded sections were visualized, captured and analysed at 12.5× to 400× magnification using an OsteoMeasure Image Analysis software v.13.2 System (Osteometrics, Atlanta, GA, USA) to determine total tissue area, tumor area and new woven bone formation in sclerotic lesions.

### Statistical analysis

Data were analysed using GraphPad Prism version 5 (GraphPad, La Jolla, CA, USA). For simple comparison of two means, Student's *t* test was performed. One-way ANOVA followed by Tukey's post-test was used when comparing three groups or more. A 2-way ANOVA followed by Bonferroni post-test was used to compare groups affected by 2 variables. *In vitro* experiments were performed three times. Results shown are from a representative experiment. Data are presented as mean ± SEM. A *p*-value < 0.05 was considered significant.
